# Step cut “V” osteotomy for acute correction in Blount’s disease treatment: A case series

**DOI:** 10.1016/j.ijscr.2019.03.044

**Published:** 2019-04-06

**Authors:** Faisal Miraj, Ajiantoro, I. Wayan Arya Mahendra Karda

**Affiliations:** a Pediatric Orthopaedics, Department of Orthopaedic and Traumatology, Fatmawati General Hospital, Faculty of Medicine Universitas, Jalan RS Fatmawati No. 1, South Jakarta, Indonesia; b Department of Orthopaedic and Traumatology, Cipto Mangunkusumo National Central Hospital, Faculty of Medicine Universitas, Indonesia

**Keywords:** Blount’s disease, Step cut “V” osteotomy, Acute correction, Outcomes

## Abstract

**Introduction:**

Severe Blount’s disease results in progressive multiplanar deformity of the lower limb. There are varus, procurvatum, and internal tibia rotational deformity. Treatment varies for each patient, depending on age, magnitude of deformity, discrepancy, psychosocial factor, and surgeon’s experience. The aim of treatment is to acutely correct deformity and realign the mechanical axis. We proposed a technique of step cut “V” Osteotomy which could achieve the aim of treatment safely and effectively.

**Methods:**

Twenty-seven legs from eighteen patients were underwent Step Cut “V” Osteotomy technique in our institution from 2015 to 2017 to produce overvalgus correction. Eleven patients had bilateral deformities, and seven patients had unilateral deformity. All patients had mechanical axis deviation (MAD) away medially to the center of the knee, increase of tibiofemoral angle, and Drennan angle. Outcomes were recorded postoperatively such as infection, compartment syndrome, nerve palsy, range of motion, stability of knee ligaments, union time, early weight bearing and recurrence.

**Results:**

From this Step Cut “V” Osteotomy, we can accurately measure the angle of correction we want to achieve preoperatively. Drennan angle and Tibiofemoral angle (TFA) of the patients improved with subsequent correction of interbal tibia rotation and procurvatum. No complication of compartment syndrome and nerve palsy were found. Patient could perform early mobilization, and weight bearing on second month after surgery because of strong fixation and good ligament stability. No significant postoperative infection occurred. Union time achieved in two to three months, but four legs from patients over 4 years old who underwent surgery experienced a recurrence.

**Conclusion:**

Step Cut "V" Osteotomy is a simple, safe and effective technique for acute correction of severe Blount’s disease. And can produce an accurate correction, high union rate and early weight bearing with no complication as a result that would be achieved at the end of treatment.

## Highlights

• Severe Blount’s disease results in progressive multiplanar deformity of the lower limb.
• Blount’s disease management should be tailored individually.
• Step Cut “V” Osteotomy developed by our institution in order to have more predictable result.
• Step Cut “V” Osteotomy provide accurate and safe correction even in severe deformity.

## 1 Introduction

Blount’s disease is a growth disorder characterized by abnormalities of the endocondral ossification of medial epiphyseal plates on the proximal tibia resulting in multiplanar deformities of the lower limbs. Inhibition of tibial epiphyseal plate activity leads to asymmetric growth resulting in deformity of varus, procurvatum (apex protruding anteriorly), and internal rotation [1,2]. The natural history of Blount’s disease leads to irreversible pathologic changes, especially at the medial portion of the proximal tibial epiphysis because of growth disturbances of the physis [3,4].

Blount’s disease management should be tailored individually based on a number of factors, such as age, classification, severity of deformity, limb length differences, psychological factors, and operator's ability and experience. The purpose of Blount’s disease therapy is to obtain lower extremities with normal joint heritability and orientation, and the same length of two limbs in skeletal maturity. Generally, children aged 2–5 years are treated with observation or trial with brace, whereas progressive deformity and older age are administered operatively [[5], [6], [7], [8], [9]].

Gradual correction with the principle of distraction osteogenesis is considered superior in the management of Blount’s disease. This is debated because the technique is believed to be safe and provides more accurate results in dealing with multiplanar deformities, including limb length differences [[6], [7], [8], [9], [10]].

In the other hand, acute correction of angular and rotational deformity in Blount’s disease can be accomplished by a proximal tibial metaphyseal osteotomy. A variety of techniques has been advocated, including closing wedge, opening wedge, dome, serrated, and inclined osteotomies [4,7,11,12].

Various difficulties encountered in Blount’s disease gradual correction can be more impacted especially for the people in Indonesia. Poor patient compliance may lead to premature consolidation of osteotomy and correction failure. In addition, the maintenance of pin sites that are less than ideal by the patient's parents in Indonesia can increase the risk of infection. Therefore, an acute correction strategy needs to be further studied to be applied for Blount’s disease patients in Indonesia. In other hand, several techniques reconstruction had been described, but easier, safer and more precise technique still demanded to be utilized for acute correction.

## 2 Methods

A cross sectional study design was used to obtain samples from 2015 to 2017 in our institution. The inclusion criteria were all Blount’s disease patient that underwent acute correction using proximal tibial osteotomy followed by internal fixation and completed the follow up for one year. Using consecutive sampling, we managed to get seventeen patients with twenty-seven extremities involved. The demographic data was taken from medical record and patient interview. The research work has been reported in line with the PROCESS criteria [13].

Among the extremities involved, all samples had a severe deformity (pre-operative Tibiofemoral Angle (TFA≥35°). All samples underwent the same surgical technique of acute correction using proximal tibial osteotomy followed by fixation with plate and screw. We used Step Cut “V” Osteotomy created by our institution inspired from modification of step cut osteotomy for cubitus varus deformity [14]. Using this simple design, we were able to correct the varus and rotational deformity while maintaining the length of the leg.

**Fig. 1:**
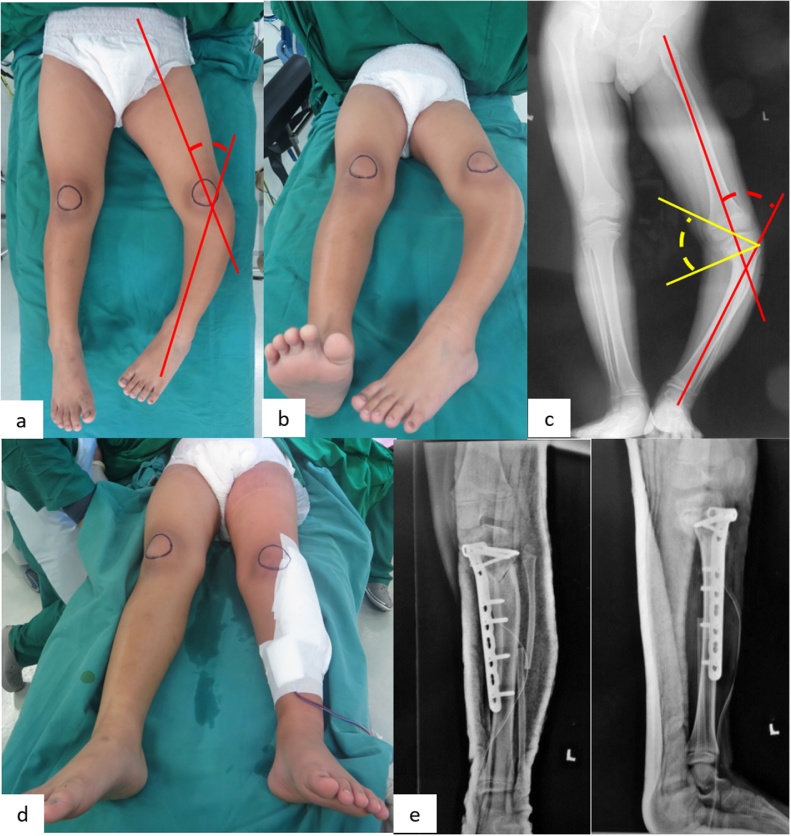
Pre and post operative results. (**a**) Pre operative clinical picture. Tibiofemoral lines from clinical picture was 46°, (**b**) Pre-operative clinical picture. Birds view, (**c**) Pre-operative Xray. Red lines – TFA 46°, Yellow lines – Drennan angle 43°, (**d**) Post operative clinical picture. Tibiofemoral lines from clinical picture was 10°, (**e**) Postoperative birds view with the deformity already corrected, (**f**) Postoperative TFA was 10°, Drennan angle was 4.3°, and from lateral xray procurvatum was corrected.

**Fig. 2:**
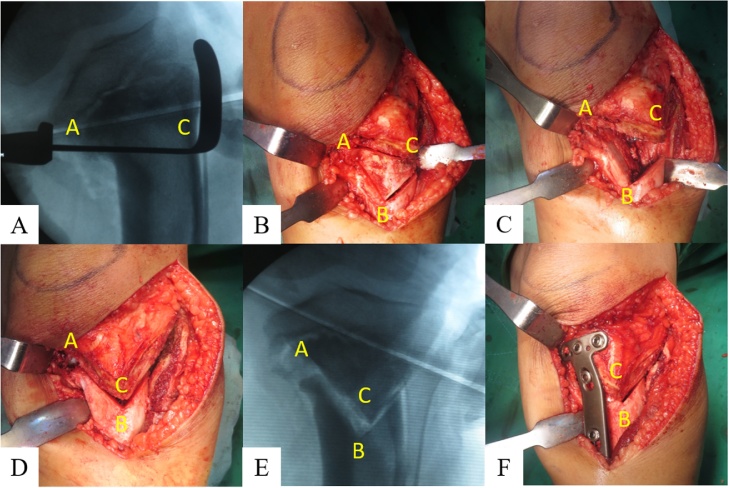
Intraoperative Step Cut “V” Osteotomy. First we did osteotomy of the fibula, and then proceeded to the Step Cut “V” Osteotomy at proximal tibia. (**a**) Guidance C-arm was used to create a horizontal line between the beak in the medial part of tibia perpendicular to the tibial axis (Line A to C). Establishment of the angle of Osteotomy correction calculated during preoperative planning **56**° at point A. Drew a straight line from point A corresponding to the angle of correction to obtain point B which was perpendicular with point C. (**b**) Triangle (Line A-B-C) which was a Step Cut “V” Osteotomy lines. (**c**) Osteotomy bone was removed, and could be used as a graft. (**d**) A rotational technique was performed where point C was rotated toward the medial so that it accumulates with point B, and point A became its hinge. (**e**) The appearance of the C-arm after the rotation was done, where it appeared that the current C point was based on the point B. (**f**) In the end, performed internal fixation with locking plate on the medial tibia, 3 proximal and 3 distal screws.

From Fig. 1, first we had to measure the preoperative TFA of patient as a reference how much osteotomy angle that we would do during the operation to get overvalgus. Example, this patient had 46° varus in preoperative TFA, so during operation we had to make 56° (46° preoperative TFA + 10° overvalgus correction) Step Cut “V” Osteotomy to get 10° valgus correction post operatively. Fig. 2 are step by step of Step Cut “V” Osteotomy.

We did one year post operative follow up to all patients (Fig. 1). We measured some of parameters such as mechanical axis deviation (MAD) based from center of the knee, TFA, Drennan angle, rate of infection, compartment syndrome, nerve palsy, the ability of weight bearing, range of motion, stability of ligaments, recurrence rate and union time.

## 3 Results

Langenskiöld system classified Blount’s disease into six stages based on the prognostic and its suitable treatment. According to the Langenskiöld classification, we divided the patients into three groups: stage I, II, and III as the mild, IV as moderate group, and stage V and VI as the severe group. From the seventeen patients (twenty seven extremities), there were nine extremities with moderate deformity, and eighteen extremities with severe deformity. The overall mean of age was 7.8 years old with the prevalence of male were more dominant. Almost all of the patients had Body Mass Index (BMI) of more than 30 which concluded as obesity. The patient’s characteristics could be seen in Table 1.

**Table 1:** Patient’s characteristics.

No.	Sex	Age (y.o)	Drennan Angle		Tibiofemoral Angle (TFA)		Mechanical axis (From the patella)		Procurvatum		Langenskiold Type	Recurrecncy	Ligament Stability	Infection	Union Rate (Months)
			Pre op	Post op	Pre op	Post op	Pre op	Post op	Pre op	Post op					
1	M	10	39 °	4 °	39 ° varus	10 ° valgus	Medial	Lateral	26 °	0°	VI	–	Stable	–	3
2	M	10	38 °	5 °	38 ° varus	10 ° valgus	Medial	Lateral	25 °	0°	VI	**+**	Stable	–	3
3	M	4	30 °	6 °	45 ° varus	10 ° valgus	Medial	Center	29 °	0°	III	–	Stable	–	2
4	M	6	51 °	8 °	50 ° varus	8 ° valgus	Medial	Center	32 °	0°	V	–	Stable	–	3
5	F	6	28 °	9 °	47 ° varus	10 ° valgus	Medial	Lateral	30 °	0°	IV	–	Stable	–	3
6	M	8	37 °	3 °	43 ° varus	10 ° valgus	Medial	Lateral	29 °	0°	IV	–	Stable	–	3
7	M	8	29 °	5 °	39 ° varus	10 ° valgus	Medial	Lateral	26 °	0°	IV	**+**	Stable	–	3
8	F	2	21 °	5 °	35 ° varus	9 ° valgus	Medial	Center	25 °	0°	III	–	Stable	–	2
9	M	7	45 °	2 °	38 ° varus	10 ° valgus	Medial	Lateral	27 °	0°	IV	–	Stable	–	3
10	F	17	39 °	3 °	42 ° varus	8 ° valgus	Medial	Center	28 °	0°	VI	–	Stable	–	4
11	M	7	43 °	4 °	46 ° varus	10 ° valgus	Medial	Lateral	30 °	0°	IV	–	Stable	–	3
12	F	8	37 °	5 °	44 ° varus	10 ° valgus	Medial	Lateral	31 °	0°	IV	–	Stable	–	4
13	F	8	43 °	2 °	48 ° varus	7 ° valgus	Medial	Center	33 °	0°	IV	–	Stable	–	4
14	F	7	27 °	4 °	36 ° varus	10 ° valgus	Medial	Lateral	29 °	0°	IV	–	Stable	–	3
15	F	8	44 °	4 °	46 ° varus	10 ° valgus	Medial	Lateral	30 °	0°	IV	–	Stable	–	4
16	F	8	56 °	4 °	61 ° varus	7 ° valgus	Medial	Center	36 °	0°	IV	–	Stable	–	4
17	M	8	28 °	4 °	35 ° varus	10 ° valgus	Medial	Lateral	26 °	0°	V	–	Stable	–	4
18	F	12	28 °	3 °	35 ° varus	10 ° valgus	Medial	Lateral	25 °	0°	V	–	Stable	–	4
19	F	6	28 °	6 °	44 ° varus	10 ° valgus	Medial	Lateral	29 °	0°	IV	–	Stable	–	3
20	F	17	38 °	4 °	40 ° varus	9 ° valgus	Medial	Lateral	28 °	0°	VI	–	Stable	–	5
21	F	8	35 °	6 °	44 ° varus	8 ° valgus	Medial	Center	29 °	0°	V	–	Stable	–	4
22	F	5	28 °	3 °	36 ° varus	10 ° valgus	Medial	Lateral	26 °	0°	IV	–	Stable	–	3
23	M	7	40 °	3 °	40 ° varus	9° valgus	Medial	Lateral	26 °	0°	IV	–	Stable	–	3
24	M	6	35 °	4 °	42 ° varus	10 ° valgus	Medial	Lateral	29 °	0°	V	**+**	Stable	–	3
25	M	9	37 °	5 °	43 ° varus	9 ° valgus	Medial	Lateral	32 °	0°	IV	**+**	Stable	–	4
26	F	4	34 °	3 °	52 ° varus	10 ° valgus	Medial	Lateral	34 °	0°	III	–	Stable	–	2
27	F	4	36 °	4 °	48 ° varus	10 ° valgus	Medial	Lateral	32 °	0°	III	–	Stable	–	2

The overall mean of pre-operative Drennan angle was 36.1°, and post-operative Drennan angle was 4.3°. The overall mean of pre-operative TFA was 42.7° varus position, and post-operative TFA was 9.4° valgus position. All of the extremities had mechanical axis at the medial to the patella in pre operation findings. After operation, most of the patients had mechanical axis at lateral to the patella, only 7 extremities in the center of patella. All of the patients had pre-operative procurvatum deformity with overall mean of 29°, and after operation they had already been corrected (0°). According to the Langenskiold classification, 4 legs classified into Langenskiold III, 14 legs classified into Langenskiold IV, 5 legs classified into Langenskiold V, 4 legs classified into Langenskiold VI. There were 4 legs from patiens over four years old having recurrency after one year of follow up (based on Drennan angle and TFA follow up). All of the patients had a good ligament stability in the intraoperative findings, and one year follow up. None of them had post-operative infection and mean of union rate was in the 2–3 months after operation.

## 4 Discussion

Blount’s disease is a common childhood condition that requires surgical correction. Either acute or gradual correction are well-established strategy with each own advantages and disadvantages. Both of those techniques are well-established treatment strategy for the late onset of Blount’s disease. While the gradual deformity correction strategy is considered to be more accurate by some authors, others stated that evidence for this was lacking, although a higher incidence of peroneal nerve palsy was recorded after acute correction which was fortunately transient. The acute correction technique provides a more practical strategy, shorter and easier monitoring, and free of pin tract infection or psychological impact that caused by large external fixator [[15], [16], [17], [18]].

Some reviews have failed to find proper case selection criteria for each correction strategy, and there was no standardization for deformity description or defining recurrence. *Khanfour* et al. [3] has proved that the acute correction was effective in less than 40° varus. Although there was a potential for neurologic injury and compartment syndrome with acute correction, but many authors still use acute correction because the general complication rate was extremely low [4].

Classification of early and late onset Blount’s disease defined by the age of less or more than four years old when the deformity started [3,4]. Although the distribution of surgical age patients are around seven years old, actually most of the patient classified as the early onset. Thus, patient that came to the hospital were most likely in a more severe deformity. This result reflected as the proportion of severe deformity group is higher than the moderate deformity group.

Conservative treatment is no longer suitable for our patients, because all of them had a moderate to severe deformity of Blount’s disease. So the proximal tibial osteotomy with acute or gradual correction was a common technique to treat this condition. We used acute correction with Step Cut “V” Osteotomy created by our institution inspired from modification of step cut osteotomy for cubitus varus deformity to provide a good alignment, and stable correction for this deformity.

Based on various techniques that have been published in the world, our technique: Step Cut “V” Osteotomy have many advantages. The first is the accuracy of the correction technique is very good, because from pre-operative planning we measured the angle of osteotomy correction wanted to be done previously based on the calculation of pre-operative TFA. Once we obtain the pre-operative TFA, we added 10° for osteotomy correction to make the position after correction 10° valgus, so we could set an accurate precision as the end result of the correction angle.

The second is that the fixation is strong enough, so that the patient can mobilize immediately. This is because the technique of osteotomy, rotation, and reconstruction that we done, produces bones that are interconnected with each other by fixation using locking plate screw. In addition, patients do not require to use cast or brace after surgery, because the correction is very stable with 3 screws on the distal and proximal fragment. And it will be more comfortable for the patients.

This study also shows correlation between pre-operative degree of deformity with post-operative Drennan angle. Drennan angle was used because it provided more representative result of tibial correction osteotomy. The Drennan angle result remains the same whether in erect or supine position and in patient with or without ligament laxity. Drennan angle of less than 11° defined as a good correctional result and Drennan angle of more than 16° has 95% chance of deformity progression [19]. In our study, all of the samples achieved post-operative Drennan angle result of less than 11° (the mean results from all patients was 4.3°) which concluded as a good result.

In the Blount’s disease itself, we can find trias deformity in patients which are varus knee, procurvatum, and internal tibia torsion. From Table 1, all patients had mechanical axis fall on the medial patella preoperatively. From post-operative findings, 7 patients had a mechanical axis fall on the center patella, and 20 patients had mechanical axis fall on the lateral patella. This technique also simultaneously corrects rotational deformities that existed in patients. And could clearly be seen from the post-operative correction results of internal tibia rotation. Not only that, the patient's procurvatum deformity had been corrected postoperatively, and both of these corrections suggested that this technique produces a good recconstruction, and accurate correction with an easy procedure.

From the 27 extremities of patients with Blount’s disease undergone Step Cut “V” Osteotomy, we found 4 patients (15%) had recurrences after one year follow up which seen from increasing TFA and Drennan angle. According to some studies, there are several factors that affect high rates of recurrence such as improper valgus correction, older surgical age, higher TFA, higher Drennan angle, and higher Langenskiold type. But from patients characteristics, four of legs from patients who had recurrency have similarity underwent surgery in the older age (over 4 years old), so we conclude that the recurrence rate was strongly influenced by the age of the patients undergoing surgery, which in theory said the age limit of under 4 years was the best age for surgery to reduce the likelihood of recurrence. The reports of recurrence rate in patient undergone surgery in more than four years old reached 55%–88% in long term follow up [[19], [20], [21]].

According to our result, none of the patients had compartment syndrome, and neurological deficits. It also confirms that the complications obtained from acute correction are quite low. We also didn’t find infection in all of patients, and the resulting ligament stability was good enough. Mean of union rate was in the 2–3 months after operation. So we can conclude acute correction in the case of Blount’s disease is quite safe to do, and resulting the stability on the knee ligaments. Referring to existing acute correction techniques, this Step Cut “V” osteotomy technique created by our institution can produce more precise correction and strong enough fixation.

## 5 Conclusion

Step Cut "V" Osteotomy is a simple, safe and effective technique for acute correction of severe Blount’s disease. And can produce an accurate correction, high union rate and early weight bearing with no complication as a result that would be achieved at the end of treatment.

## Conflicts of interest

The authors have no ethical conflicts to disclose.

## Funding

There is no sources of funding sponsor in this manuscript.

## Ethical approval

The authors have no ethical conflicts to disclose.

## Consent

On behalf of the patient, their parents or guardians have given consent to be enrolled in this study.

## Author contribution

1. Faisal Miraj, MD. Contributed as making the study design, funding, analyzing the data and final approval of manuscript.

2. Ajiantoro, MD. Contributed as making the study design, collecting, and analyzing the data, and writing manuscript.

3. I Wayan Arya Mahendra Karda, MD. Contributed as analyzing the data and writing manuscript.

## Registration of research studies

The name of the registry: researchregistry.com.

UIN: researchregistry4738.

## Guarantor

Faisal Miraj, MD.

## Provenance and peer review

Not commissioned, externally peer-reviewed.
